# FLI1 mediates the selective expression of hypoxia‐inducible factor 1 target genes in endothelial cells under hypoxic conditions

**DOI:** 10.1002/2211-5463.13220

**Published:** 2021-06-26

**Authors:** Guodan Zeng, Tao Wang, Jingyao Zhang, Y. James Kang, Li Feng

**Affiliations:** ^1^ Key Laboratory of Transplant Engineering and Immunology Ministry of Health West China Hospital Sichuan University Chengdu China; ^2^ Regenerative Medicine Research Center West China Hospital Sichuan University Chengdu China; ^3^ Memphis Institute of Regenerative Medicine University of Tennessee Health Science Center Memphis TN USA

**Keywords:** FLI1, hypoxia, hypoxia‐inducible factor, transcription target gene

## Abstract

The selective expression of hypoxia‐inducible factor (HIF) target genes in different physiological and pathological environments forms the basis for cellular adaptation to hypoxia in development and disease. Several E26 transformation‐specific (ETS) transcription factors have been shown to specifically regulate the expression of a subset of HIF‐2 target genes. However, it is unknown whether there are ETS factors that specifically regulate hypoxia‐induced HIF‐1 target genes. The present study was undertaken to explore whether friend leukemia integration 1 (FLI1), an ETS transcription factor, regulates the expression of HIF‐1 target genes. To investigate this possibility, EA.hy926 cells were exposed to 20% O_2_ (normoxia) or 1% O_2_ (hypoxia). Western blotting, immunofluorescence staining, and RT‐qPCR revealed that FLI1 mRNA and protein levels increased slightly and that the FLI1 protein co‐localized with HIF‐1α in the nucleus under hypoxic conditions. Further analysis showed that, in the absence of FLI1, the hypoxia‐mediated induction of HIF‐1 target genes was selectively inhibited. The results from immunoprecipitation and luciferase reporter assays indicated that FLI1 cooperates with HIF‐1α and is required for the transcriptional activation of a subset of HIF‐1 target genes with a core promoter region containing FBS in proximity to a functional hypoxia response element (HRE). Furthermore, ChIP analysis further confirmed the direct interaction between FLI1 and the promoter region of FLI1‐dependent HIF‐1 target genes under hypoxia. Together, this study demonstrates that FLI1 is involved in the transactivation of certain HIF‐1 target genes in endothelial cells under hypoxic conditions.

## Graphical abstract

In this work, we found that FLI1 co‐regulates the expression of certain HIF‐1 target genes by directly binding to the promoter region containing FBS in proximity to the core hypoxia response element (HRE). This work provides new mechanistic insights into the fine‐tuning of HIF‐1 signaling in endothelial cells under hypoxic conditions and the development of cancer.

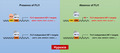

**Abbreviations**
BNIP3: BCL2 interacting protein 3
DDIT4: DNA damage‐inducible transcript 4
EBS: ETS factor binding site
ETS: E26 transformation‐specific
FLI1: friend leukemia integration 1
GO: Gene Ontology
HIF: hypoxia‐inducible factor
HRE: hypoxia response element
PGK1: phosphoglycerate kinase 1
siControl: mismatch control siRNA
siFLI1: FLI1‐specific siRNA
SLC2A1: solute carrier family 2 member 1
VEGFA: vascular endothelial growth factor A

Hypoxia is an imbalance between oxygen supply and demand that occurs in embryonic development, as well as in pathological situations such as cancer, ischemic heart disease, and wound healing [1, 2, 3, 4]. To cope with decreased oxygen concentrations, a broad range of genes involved in angiogenesis, glucose metabolism, erythropoiesis, and apoptosis are transcriptionally activated, primarily by hypoxia‐inducible factors (HIFs) [5, 6]. HIFs are heterodimeric transcription factors composed of an oxygen‐sensitive α subunit (HIF‐α) and a constitutively expressed β subunit (HIF‐β) [7, 8]. Under normoxic conditions, HIF‐α is hydroxylated by prolyl hydroxylases (PHDs) and subsequently degraded via the ubiquitin‐proteasome pathway. Under hypoxic conditions, HIF‐α is stabilized and forms a transcriptional complex with HIF‐β and other co‐factors, such as E1A binding protein p300 (p300) and CREB‐binding protein (CBP). The HIF‐α/β complex subsequently initiates the transcription of target genes by binding to the hypoxia response element (HRE, 5′‐G/ACGTG‐3′) in their promoter regions. Hundreds of genes are known to be directly transactivated by HIFs [9, 10]. Interestingly, the expression profile of HIF target genes under hypoxic conditions exhibits cell type‐dependent differences, as well as differences the correlate with the magnitude and timing of hypoxia [11, 12]; however, the molecular mechanisms that confer target gene specificity remain unclear.

Several studies have suggested that other transcription factors activated by hypoxia may recognize and bind to specific DNA elements in the promoter regions of HIF target genes, thereby regulating HIF target gene selectivity [13, 14, 15, 16, 17]. In particular, members of the E26 transformation‐specific (ETS) family of transcription factors continue to be identified as playing important roles in regulating HIF transcriptional activity under hypoxic conditions [18, 19]. ETS proto‐oncogene 1 (ETS1) is the first member of the ETS family of transcription factors that was demonstrated to interact directly with HIF‐2α and was shown to promote the activation of vascular endothelial growth factor receptor 2 (*FLK1*) in endothelial cells under hypoxic conditions [20]. Other ETS family transcription factors, such as ETS proto‐oncogene 2 (ETS2) and ETS transcription factor ELK1 (ELK1), are also required for hypoxic induction of a subset of HIF‐2 target genes in endothelial cells and cancer cells [21, 22]. Analysis of the promoter regions of these HIF‐2 specific targets reveals that there is at least one putative ETS factor binding site (EBS) containing the consensus sequence 5′‐GGAA/T‐3′ near the core HRE in the promoter region [21]. Taken together, these findings suggest that ETS transcription factors may function as transcriptional co‐regulators for certain HIF‐2 target genes by interacting with HIF‐2α and mediating simultaneous binding of these two factors to adjacent DNA elements in the promoter.

Previous studies have demonstrated the necessary role of certain ETS transcription factors in activating the expression of HIF‐1 regulated pro‐angiogenic genes in endothelial cells, such as vascular endothelial growth factor A (*VEGFA*) [23], vascular endothelial growth factor receptor 1 (*FLT1*) [24], angiopoietin‐2 (*ANGPT2*) [25], and endothelial nitric oxide synthase (*NOS3*) [26]; it seems reasonable to propose that ETS transcription factors may also contribute to HIF‐1 target gene selectivity under hypoxic conditions.

Recently, we demonstrated the presence of putative friend leukemia integration 1 (FBSs, 5′‐C[CA]GGAAGT‐3′) in the promoter regions of a subset of HIF‐1 target genes [27]. FLI1 is an endothelial‐specific ETS transcription factor that is essential for vascular development [28]. Importantly, FLI1 has been shown to regulate the transcription of several vascular homoeostasis genes that are preferentially regulated by HIF‐1, such as endoglin (*ENG*) and heme oxygenase 1 (*HMOX1*) [29, 30]. This suggests that FLI1 may function as a HIF‐1α co‐regulator in endothelial cells to selectively induce the expression of specific HIF‐1 target genes under hypoxic conditions.

In this study, we found that FLI1 expression is slightly increased in a human umbilical vein endothelial cell line (EA.hy926) after exposure to hypoxic conditions. Abolishing FLI1 expression significantly inhibited the hypoxia‐induced expression of several, but not all, HIF‐1 target genes, such as BCL2 interacting protein 3 (*BNIP3*), phosphoglycerate kinase 1 (*PGK1*), solute carrier family 2 member 1 (*SLC2A1*), and *VEGFA*. Further analysis revealed that FLI1 physically interacts with HIF‐1α in the nucleus and promotes the transcription of these FLI1‐dependent HIF‐1 targets by binding to the FBS in close proximity to the HRE in the promoters of these genes under hypoxic conditions. Taken together, our findings suggest that FLI1 may be an important regulator responsible for proper HIF‐1 target gene expression in endothelial cells under hypoxic conditions.

## Materials and methods

### Cell culture and treatments

The EA.hy926 and HEK293T cell lines were obtained from American Type Culture Collection (ATCC) and cultured in high‐glucose Dulbecco's modified Eagle's medium (H‐DMEM, GIBCO, Grand Island, NY, USA) supplemented with 10% fetal bovine serum (Natocor, Cordoba, Argentina) and 1% penicillin/streptomycin at 37°C in a 5% CO_2_ incubator. Cells were maintained in a tri‐gas incubator (ESCO, SGP) with 1% O_2_ for 8 h or 16 h to induce hypoxia after achieving 80%‐90% confluence.

### Western blotting

Cells were harvested using RIPA lysis buffer containing a protease inhibitor cocktail (Roche, Mannheim, Germany) after washing twice with ice‐cold PBS. The protein concentration was measured using a BCA Protein Assay Kit (Thermo Pierce, Rockford, IL, USA). Equal amounts of protein (30 μg) from each sample were separated on a SDS/polyacrylamide electrophoresis gel and transferred to polyvinylidene fluoride (PVDF) membranes (Bio‐Rad, Hercules, CA, USA). After blocking with 5% nonfat milk for 1 h at room temperature, the PVDF membranes were incubated at 4 °C overnight in blocking solution containing the following antibodies: goat anti‐human HIF‐1α polyclonal antibody (AF1935, R&D Systems, Minneapolis, MN, USA, 1 : 1000), rabbit anti‐human FLI1 polyclonal antibody (ab15289, Abcam, Cambridge, UK, 1 : 1000), rabbit anti‐human polyclonal SLC2A1 antibody (A11727, ABclonal, Wuhan, China, 1 : 1000), mouse anti‐human monoclonal DDIT4 antibody (67059‐1‐Ig, Proteintech, Rosemont, IL, USA, 1 : 1000), mouse anti‐human β‐actin monoclonal antibody (AY0573, Abways, Shanghai, China, 1 : 1000), and mouse anti‐human GAPDH monoclonal antibody (TA‐08, ZSGB‐BIO, Beijing, China, 1 : 1000). The membranes were then incubated with appropriate secondary antibodies for 1 h at 37 °C after washing in TBST buffer three times. Target proteins were visualized using an ECL chemiluminescent detection system (FUSION FX, Vilber Lourmat, Marne‐la‐Vallée, France) and analyzed by densitometry using fusion software (Vilber Lourmat, Marne‐la‐Vallée, France).

### RT‐qPCR analysis

Total RNA was extracted from cultured cells using TRIzol Reagent (Invitrogen, Carlsbad, CA, USA), and 1 μg of the extracted RNA was reverse transcribed to cDNA using a PrimeScript RT Reagent Kit (TaKaRa, Kusatsu, Japan). Real‐time qPCR was performed using TB Green Premix Ex Taq II (TaKaRa, Kusatsu, Japan) and a Thermo Q6 Real‐Time System according to the manufacturer's instructions. Gene expression relative to that of *β‐actin* was analyzed for each sample using the 2^−ΔΔCt^ method, as previously described [31]. The primer sequences are shown in Table 1.

**Table 1:** RT‐qPCR primer sequences.

Primer		Forward sequence (5′–3′)	Reverse sequence (5′–3′)
FLI1		GTGAGGACGTGCAGGGAA	TCCCGAGACGCTCAGCTC
HIF‐1α		GTCTGAGGGGACAGGAGGAT	CTCCTCAGGTGGCTTGTCAG
BNIP3		TCAGCATGAGGAACACGAGCGT	GAGGTTGTCAGACGCCTTCCAA
PGK1		CTCCGCTTTCATGTAGAGGAAG	GACATCTCCTAGTTTGGACAGTG
SLC2A1		CTGAAGTCGCACAGTGAATA	TGGGTGGAGTTAATGGAGTA
VEGFA		TTGCCTTGCTGCTCTACCTCCA	GATGGCAGTAGCTGCGCTGATA
DDIT4		CACTGGCTTCCGAGTCATCA	TATTCCCCCACCTCCACCTT
ADM		ATGAAGCTGGTTTCCGTCG	GACATCCGCAGTTCCCTCTT
ANKRD37		TTAGGAGAAGCTCCACTACACAA	CACTGGCTACAAGCAGGCT
KDM3A		GTGCTCACGCTCGGAGAAA	GTGGGAAACAGCTCGAATGGT
β‐actin		CCACGAAACTACCTTCAACTCC	GTGATCTCCTTCTGCATCCTGT

### Immunofluorescence

Cells grown on microscope slides were fixed in 4% paraformaldehyde for 10 min and permeabilized with 0.1% Triton X‐100 for 10 min at room temperature, followed by blocking with 2% bovine serum albumin (BSA, Gentihold, Beijing, China) for 1 h at 37 °C. The slides were then incubated in 2% BSA containing a goat anti‐human HIF‐1α polyclonal antibody (AF1935, R&D Systems, Minneapolis, MN, USA, 1 : 100) and a rabbit anti‐human FLI1 polyclonal antibody (ab15289, Abcam, Cambridge, UK, 1 : 100) overnight at 4 °C, followed by incubation with Alexa Fluor® 488 donkey anti‐goat (Thermo Fisher, Carlsbad, CA, USA) or Alexa Fluor® 568 goat anti‐rabbit (Thermo Fisher, Carlsbad, CA, USA) secondary antibody at 37 °C for 30 min. DAPI was used to stain the cell nuclei. Cells that were stained with PBS instead of the primary antibody served as a negative control. Fluorescent cell images were obtained by confocal microscopy (Nikon, Tokyo, Japan).

### FLI1 gene silencing

Cells cultured in 6‐well plates were transfected with 50 nm of a human FLI1‐specific siRNA (siFLI1‐1 or siFLI1‐2) or a mismatch control siRNA (siControl) using Lipofectamine 3000 Reagent (Thermo Fisher, Carlsbad, CA, USA) according to the manufacturer's instructions. The FLI1‐specific siRNAs were designed by TSINGKE (Beijing, China). The sequences were as follows: siFLI1‐1, sense 5′‐GAUUGAGUGUCAAAGAAGA‐3′ and antisense 5′‐UCUUCUUUGACACUCAAUC‐3′; siFLI1‐2, sense 5′‐CCGUUAUUACUAUGAUAAA‐3′, and antisense 5′‐UUUAUCAUAGUAAUAACGG‐3′.

### Luciferase reporter assay

The previously validated core promoter sequences from *BNIP3* (−635/−16, 620bp), *VEGFA* (−1460/−861, 600bp), and *DDIT4* (−197/+298, 495bp) were cloned into separate pGL3‐Basic firefly luciferase reporter plasmids (Promega, Madison, WI, USA; see details in Fig. 4) [32, 33, 34]. Next, HEK293T cells were transfected with a FLI1‐specific siRNA (siFLI1‐1) or a mismatch control siRNA (siControl) for 24 h, followed by co‐transfection with 1 µg of one of the firefly luciferase plasmids and 20 ng of a Renilla internal control luciferase plasmid (pRL‐SV40, Promega, Madison, WI, USA) using Lipofectamine 3000 Reagent (Thermo Fisher, Carlsbad, CA, USA), for 32 h. The cells were then cultured with or without 1% O_2_ for 16 h. Luciferase activities were measured using a Dual‐Luciferase Reporter Assay Kit (Promega, Madison, WI, USA) according to the manufacturer's instructions.

### ChIP assay

Cells were fixed with 1% formaldehyde (Sigma, Haverhill, MA, USA) at room temperature for 10 min, and the reaction was terminated by glycine (Sigma, Haverhill, MA, USA) at a final concentration of 0.125 m. Cells were scraped from culture flasks after being washed three times with ice‐cold PBS. Nuclei were isolated and lysed, and DNA in the lysate was interrupted to lengths between 200 and 800 bp by ultrasonic treatment (Fig. S3B). Sheared DNA was incubated with 20 μL Dynabeads Protein G beads (Invitrogen, Carlsbad, CA, USA) at room temperature for 20 min first to remove nonspecific binding The antibody–beads complex was prepared at room temperature for 30 min as follows: 40 μL Dynabeads Protein G beads were incubated with 5 μg mouse anti‐human FLI1 monoclonal antibody (MA1‐196, Invitrogen, Carlsbad, CA, USA) or homologous IgG with gentle rotation. IgG was served as a negative control. 10% sheared DNA was removed as ‘input’ DNA; then, equal amounts of DNA from each sample were incubated with antibody–beads complexes at 4 °C overnight. After immunoprecipitation, the nonspecific binding DNA was removed by washing buffer as described in previous study [35], and the FLI1‐binding DNA was eluted by elution buffer (1% SDS, 0.1 m NaHCO_3_). The DNA product was incubated with ribonuclease (TaKaRa, Kusatsu, JPN, 0.2 mg·mL^−1^) at 37 °C for 1 h and followed by protease K (Roche, Mannheim, Germany) at 42 °C overnight. Purified DNA was detected by RT‐qPCR, and the binding level was analyzed relative to IgG level using the 2^−ΔΔCt^ method in each sample. The primer sequences for ChIP are shown in Table 2.

**Table 2:** Primer sequences for ChIP‐qPCR.

Primer	Forward sequence (5′–3′)	Reverse sequence (5′–3′)
BNIP3‐1	GAGCCTCCGCTTCTTCCTGC	CGCCCCTGCGTGAACAGC
BNIP3‐2	GGCCGCTTCCCTGCACGTC	GCCGGGTTCTCCTTTGAAGGG
BNIP3‐3	CCGTGGTAGCCAGTGCCC	GACCGCCTGAGGTGAGCC
BNIP3‐NC	GAGAACCCACAGAAACGG	CCCACTAAATAGCCCACC
VEGFA‐1	ATAGCCAGGTCAGAAACCA	TCCCTAAGTGCTCCCAAA
VEGFA‐2	CAACAGGTCCTCTTCCCTCC	CCTCTGACAATGTGCCATCT
VEGFA‐NC	TTGCCTTGCTGCTCTACCTCCA	GATGGCAGTAGCTGCGCTGATA

### Co‐immunoprecipitation

Cells were treated with NP40 Cell Lysis Buffer (Invitrogen, Carlsbad, CA, USA) containing a protease inhibitor cocktail (Roche, Mannheim, Germany) and PMSF (1 mm, Sigma, Haverhill, MA, USA) after being washed three times with ice‐cold PBS and were then scraped off of the cell culture dishes. The cell pellets were resuspended by vortexing 3–4 times for 5 min each time. The protein concentration was determined using a BCA Protein Assay Kit (Thermo Pierce, USA). Protein samples (1 mg) were immunoprecipitated with 1 μg of a mouse anti‐human FLI1 monoclonal antibody (MA1‐196, Invitrogen, Carlsbad, CA, USA) or homologous IgG overnight at 4 °C with gentle rotation. The immune complexes were collected with Dynabeads Protein A beads (50 µL, Invitrogen, Carlsbad, CA, USA), washed, boiled in SDS/PAGE Loading Buffer (Beyotime, Shanghai, China) at 100 °C for 5 min, and then subjected to western blot analysis.

### RNA sequencing (RNA‐seq) and data analysis

Cells were collected using TRIzol Reagent (Invitrogen, Carlsbad, CA, USA) after 8 h of incubation under normoxic (20% O_2_) or hypoxic (1% O_2_) conditions. RNA quantity and quality were measured using a NanoPhotometer spectrophotometer (IMPLEN, Los Angeles, CA, USA), and RNA integrity was measured using a RNA Nano 6000 Assay Kit and a Bioanalyzer 2100 system (Agilent Technologies, Palo Alto, CA, USA). Sequencing libraries were generated using the NEBNext UltraTM RNA Library Prep Kit for Illumina (NEB, Ipswich, MA, USA) according to the manufacturer's recommendations, and index codes were assigned to the sequences to each sample. Library quality was assessed using an Agilent Bioanalyzer 2100 system (Agilent Technologies, Palo Alto, CA, USA). Finally, the libraries were sequenced on an Illumina Novaseq platform, and 150‐bp paired‐end reads were generated. The sequencing data were evaluated and filtered using FastQC. Reads containing adapters or ploy‐N sequences and low‐quality reads were filtered out. An index based on the human reference genome (GRCh38) was built using Hisat2 v2.0.5, and paired‐end clean reads were aligned to the human reference genome using Hisat2 v2.0.5.

Pearson correlation analysis was performed using the cor function in R, and one replicate from the normoxia group (Nor_3) was excluded to obtain high‐confidence reads (Fig. S1A). Differential expression analysis was performed using the DESeq2 R package v1.16.1 (|log2FC| > 1, *P* value < 0.05). Gene Ontology (GO) enrichment analysis of differentially expressed genes was performed using the clusterprofiler r package, which corrected for gene length bias. GO terms with corrected *P* value < 0.05 were considered to be significantly differentially expressed genes.

### Measurement of glucose and lactate

Cells were treated as described previously. Glucose (Applygen Technologies, Beijing, China) and lactate (Nanjing Jiancheng Bioengineering Institute, Nanjing, China) assay kits were used to measure glucose consumption and lactate production in the supernatants of culture medium following the manufacturer's instructions.

### Statistical analysis

All data were presented as mean ± SD and analyzed by spss Statistics 26 (IBM, Chicago, IL, USA) and graphpad prism 8.0 (GraphPad Software, San Diego, CA, USA) computer programs. The normal distribution and equal variances of the data were tested by Shapiro–Wilk test and Levene's test prior to Student's unpaired *t*‐test or two‐way ANOVA analysis, respectively. If the data were not equally distributed, then data were log_10_ transformed prior to running statistical analysis. At least three independent experiments were carried out, and *P* value < 0.05 was considered significant.

## Results

### FLI1 expression is induced in the nucleus under hypoxic conditions

**Fig. 1:**
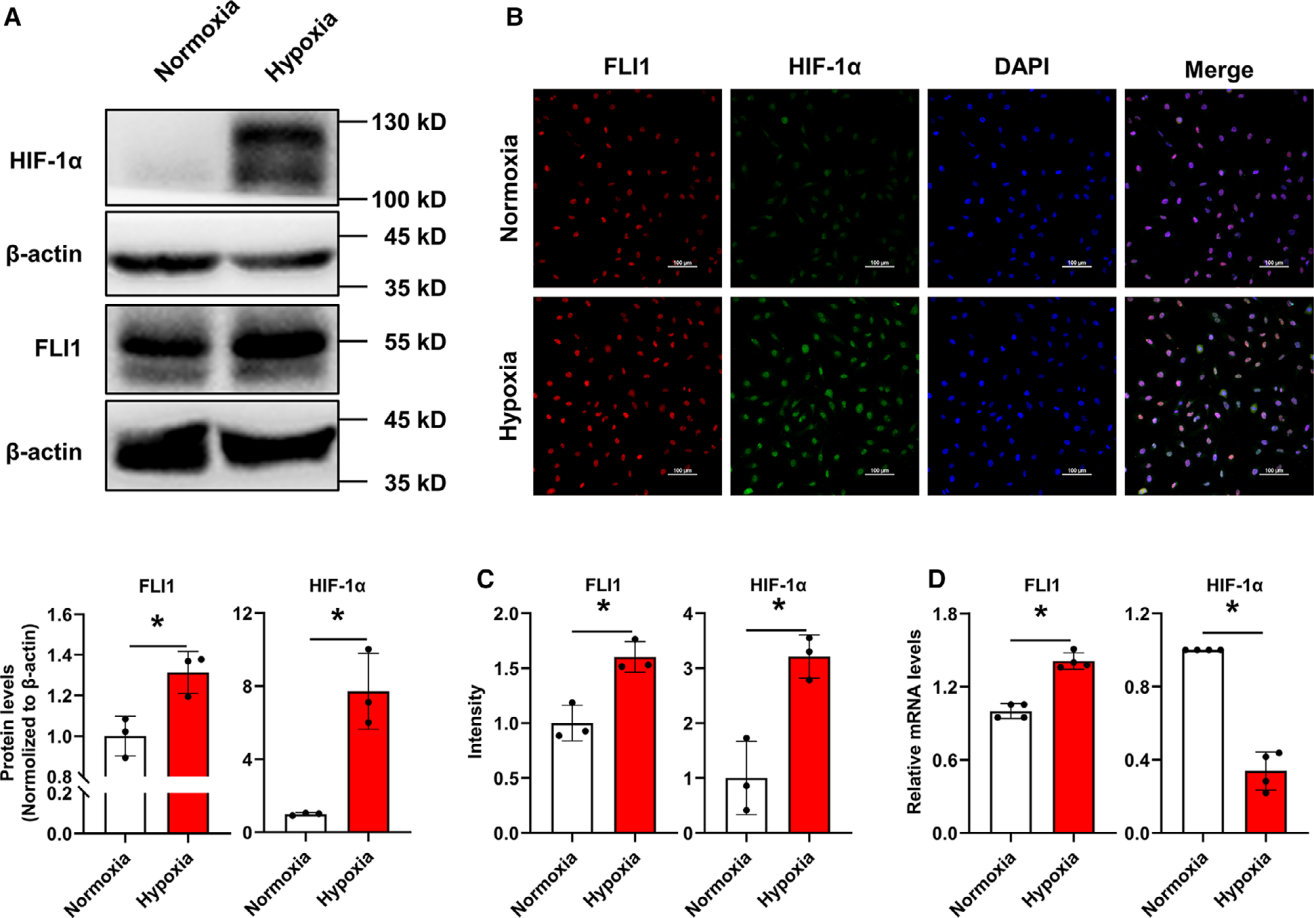
Hypoxia induces FLI1 and HIF‐1α expression in EA.hy926 cells. EA.hy926 cells were exposed to 20% O_2_ (normoxia) or 1% O_2_ (hypoxia) for 8 h. (A) Changes in FLI1 and HIF‐1α expression were evaluated by western blotting. The immunoblots are shown in the upper panel, and the quantitative data are shown in the lower panel. β‐actin served as a loading control. (B) The subcellular localization of FLI1 and HIF‐1α was examined by immunofluorescence staining. Red, green, and blue staining indicates FLI1, HIF‐1α, and the nuclear marker DAPI, respectively. Two to three visual fields were assessed for each slide. Scale bar, 100 μm. (C) The relative intensity of the FLI1 and HIF‐1α fluorescence signals was quantified using imagej software. (D) Changes in *FLI1* and *HIF‐1α* mRNA expression levels were measured by RT‐qPCR. Data are presented as mean ± SD, *n* = 3–4. *P < 0.05, as determined by Student's unpaired *t*‐test against the normoxia group.

We first examined changes in FLI1 expression under hypoxic conditions in an endothelial cell line, EA.hy926. As determined by western blotting, treating cells with 1% O_2_ for 8 h caused a significant increase in HIF‐1α expression and a slight increase in FLI1 expression (Fig. 1A). Immunofluorescence analysis confirmed that the expression of both HIF‐1α and FLI1 was increased in these cells and that these two proteins co‐localized in the nucleus after exposure to hypoxic conditions (Fig. 1B,C). Moreover, FLI1 mRNA levels were also slightly increased, while HIF‐1α mRNA levels were decreased, in response to hypoxia (Fig. 1D), indicating transcriptional regulation of FLI1 and post‐translational modification of HIF‐1α under hypoxic conditions.

### FLI1 gene silencing selectively inhibits hypoxia‐induced activation of HIF‐1 signaling

**Fig. 2:**
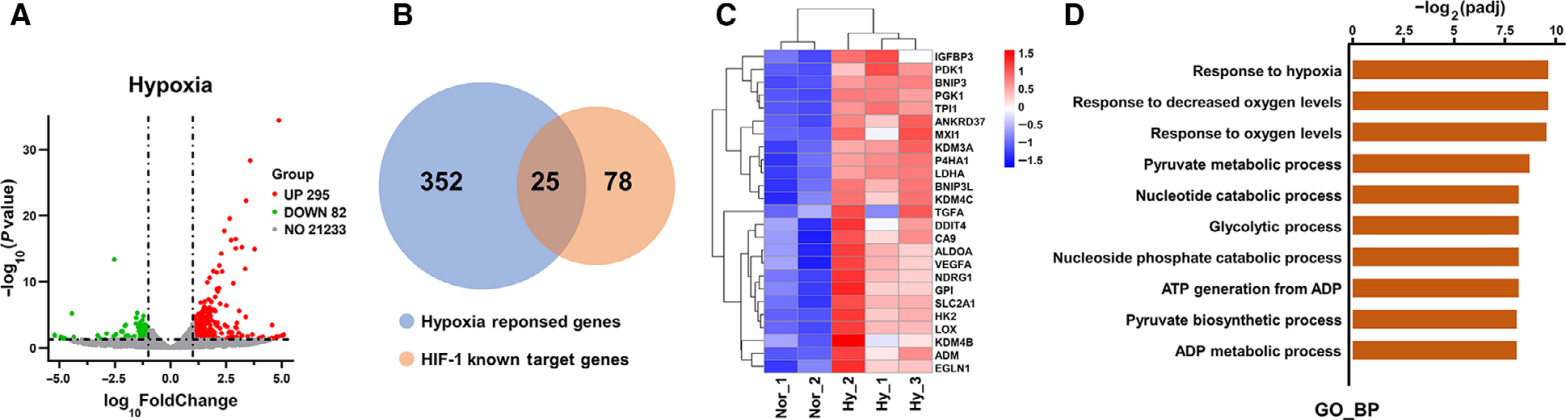
The expression profile of HIF‐1 target genes activated in EA.hy926 cells after exposure to hypoxic conditions was evaluated by RNA sequencing. RNA sequencing analysis was performed to identify HIF‐1 target genes whose expression in EA.hy926 cells changed significantly after 8 h of hypoxia. (A) Volcano plot of differentially expressed genes (DEGs) between the hypoxia group and the normoxia group. Gray dots represent genes that were not differentially expressed, red dots represent up‐regulated genes, and green dots represent down‐regulated genes. |Log2FC| > 1, *P* < 0.05. (B) A Venn diagram showing the overlap between the DEGs identified here and previously validated HIF‐1 target genes. (C) Heatmap and (D) GO_BP analysis of the 25 genes in the overlapping region of the Venn diagram shown in (B).

Next, we performed RNA sequencing to profile activated HIF‐1 target genes in EA.hy926 cells after 8 h of hypoxia. Transcriptome analysis identified a total of 377 differentially expressed genes (DEGs) (|log2FC| > 1, *P* < 0.05) under hypoxic conditions compared with normoxia group, including 295 up‐regulated genes that were mainly involved in glucose and energy metabolism and 82 down‐regulated genes that did not map to any unique cellular pathway or process (Figs 2A and Fig. S1). Comparison with a previously validated HIF‐1 target gene list [36] showed that 25 HIF‐1 targets were up‐regulated by hypoxia in EA.hy926 cells (Fig. 2B,C and Table S1). Notably, these up‐regulated HIF‐1 target genes were mainly related to glucose and energy metabolism (Fig. 2D).

**Fig. 3:**
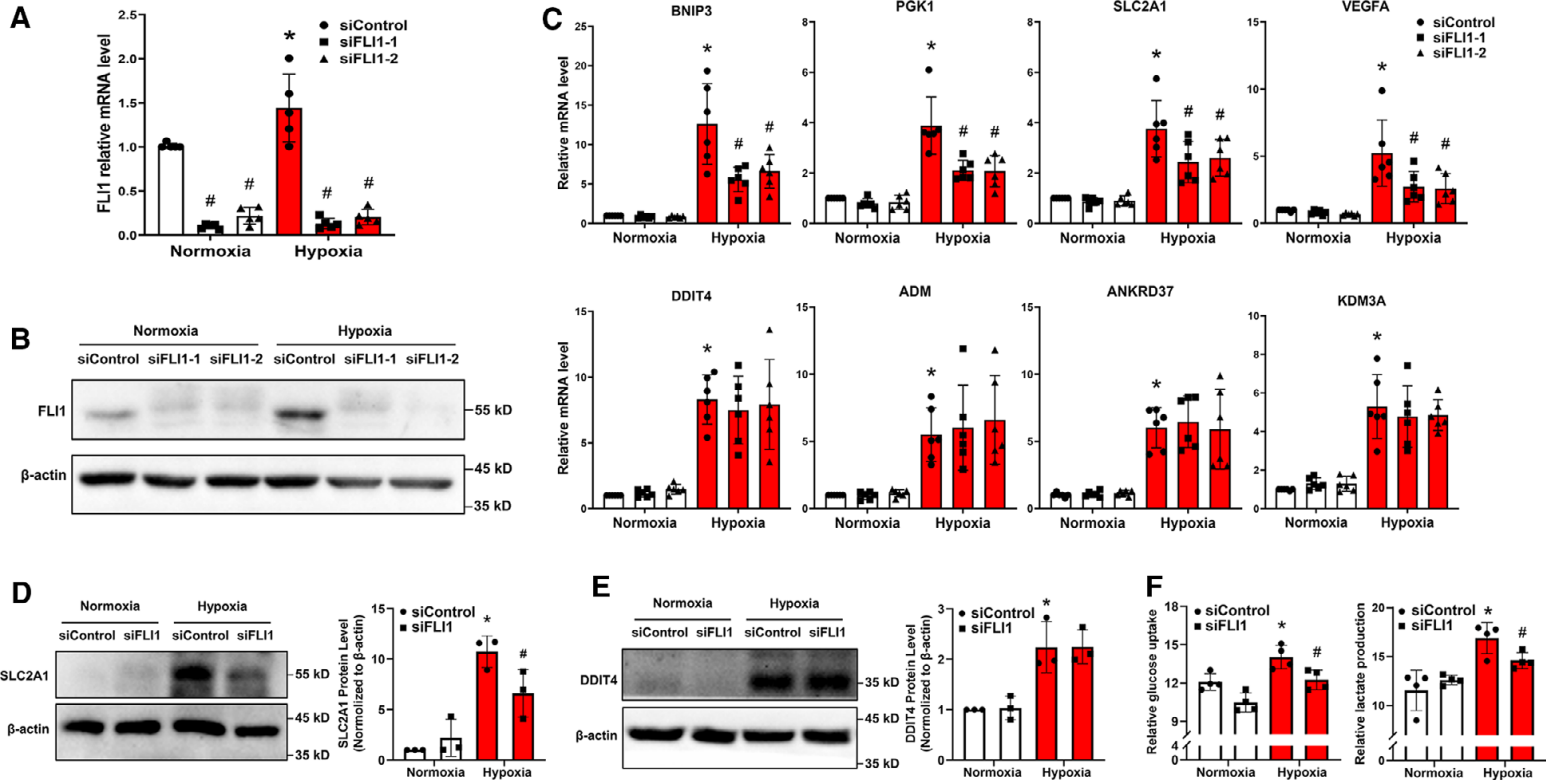
FLI1 regulates the expression of a subset of HIF‐1 target genes in EA.hy926 cells under hypoxic conditions. EA.hy926 cells were transfected with either a mismatch control siRNA (siControl) or a FLI1‐specific siRNA (siFLI1‐1 or siFLI1‐2) for a total of 48 h, with or without the final 8 h spent under hypoxic conditions. (A, B) The gene silencing efficiency of the two FLI1‐specific siRNAs was evaluated by RT‐qPCR (A) and western blotting (B). (C) *BNIP3*, *PGK1*, *SLC2A1*, *VEGFA*, *DDIT4*, *ADM*, *ANKRD37*, and *KDM3A* mRNA levels in the presence or absence of FLI1 under normoxic or hypoxic conditions were measured by RT‐qPCR. (D, E) SLC2A1 and DDIT4 protein levels were detected by western blotting. (F) Glucose and lactate concentrations were tested in the culture medium using glucose and lactate assay kits. The data are presented as the mean ± SD, *n* = 3–6. *P < 0.05 compared with the normoxia siControl group, ^#^ *P* < 0.05 compared with the corresponding siControl group, as determined by two‐way ANOVA analysis.

To investigate whether FLI1 is involved in regulating hypoxia‐induced HIF‐1 target gene expression, we abolished FLI1 expression using siRNA and examined the changes in the expression of HIF‐1 target genes under normal or hypoxic conditions. Based on the RNA‐seq data, we selected the eight HIF‐1 target genes whose expression changed most significantly (Log2FC > 2, *P* < 0.0001) after 8 h of hypoxia for further examination, including *BNIP3*, *PGK1*, *SLC2A1*, *VEGFA*, DNA damage‐inducible transcript 4 (*DDIT4*), adrenomedullin (*ADM*), ankyrin repeat domain 37 (*ANKRD37*), and lysine demethylase 3A (*KDM3A*). FLI1 gene silencing significantly reduced FLI1 mRNA and protein expression under both normoxic and hypoxic conditions (Fig. 3A,B). Interestingly, knockdown of FLI1 specifically inhibited the hypoxia‐induced activation of *BNIP3*, *PGK1*, *SLC2A1*, and *VEGFA* expression, but had no effect on the expression of the other four HIF‐1 target genes tested (*DDIT4*, *ADM*, *ANKRD37*, and *KDM3A*; Fig. 3C). Furthermore, by examining the protein levels of two representative HIF‐1 targets, *SLC2A1* and *DDIT4*, we observed a significant reduction of the protein levels of SLC2A1, but not DDIT4, after FLI1 knockdown under hypoxia (Fig. 3D,E), which was consistent with the expression pattern of their mRNA levels.

Given FLI1 is involved in the regulation of HIF‐1 target glucose metabolic genes, such as *PGK1* and *SLC2A1*, we thus investigated whether FLI1 knockdown would have any effect on the cellular glucose metabolism by measuring the change in glucose uptake and lactate production in endothelial cells under hypoxic conditions. As shown in Fig. 3F, FLI1 deletion significantly inhibited the hypoxia‐induced glucose uptake and lactate production. Taken together, these results indicate that FLI1 is involved in cellular hypoxic response, such as metabolic adaptation, at least in part by selectively regulating HIF‐1 signaling pathway.

### FLI1 cooperates with HIF‐1α to activate the transcription of HIF‐1 target genes

**Fig. 4:**
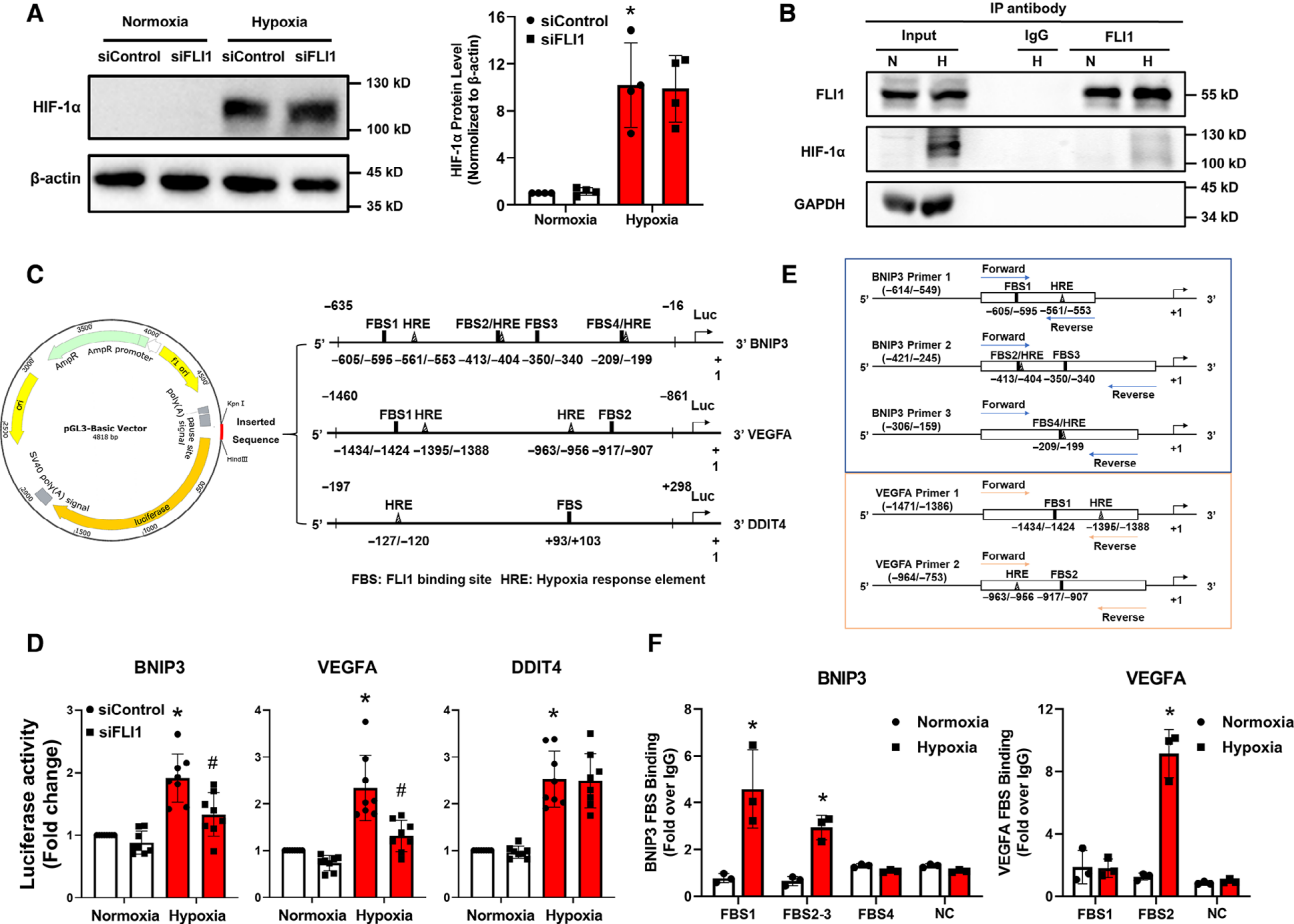
FLI1 interacts with HIF‐1α and selectively regulates HIF‐1 transcriptional. (A) HIF‐1α protein level in the absence of FLI1 was detected. Cells treated as described in Fig. 3. (B) Co‐immunoprecipitation of proteins from EA.hy926 cells with or without exposure to 16 h of hypoxia was performed using an anti‐IgG or anti‐FLI1 antibody, and then, FLI1 and HIF‐1α proteins were detected by western blotting. Anti‐GAPDH antibody that served as a negative control. (C) Schematic diagram of the construction of the *BNIP3*, *VEGFA,* and *DDIT4* luciferase reporter vectors. The core promoter sequences of *BNIP3*, *VEGFA*, or *DDIT4* containing functional HREs and putative FBSs were inserted into individual pGL3‐basic vectors. (D) HEK293T cells were transfected with either a mismatch control siRNA (siControl) or a FLI1‐specific siRNA (siFLI1) for 24 h, following by a 48 h of transfection with the luciferase plasmids, with or without the final 16 h spent under hypoxic conditions. Luciferase reporter assay analysis of the luciferase activities of the *BNIP3*, *VEGFA,* and *DDIT4* promoters. (E) Schematic diagram of ChIP‐qPCR specific primers for *BNIP3* and *VEGFA*. (F) EA.hy926 cells were exposed or not to 16 h of hypoxia. Determination by ChIP‐qPCR of the potential FLI1 binding sites of the *BNIP3* promoter, and the *VEGFA* promoter. Data are presented as the mean ± SD, *n* = 3–8. *P < 0.05 compared with the normoxia siControl group (A, D) or normoxia group (F), ^#^ *P* < 0.05 compared with the hypoxia siControl group, as determined by two‐way ANOVA analysis (A, D) or Student's unpaired *t*‐test (F).

To the regulatory mechanism underlying FLI1‐mediated selective expression of HIF‐1 target genes, we first examined the change of HIF‐1α protein levels in the absence of FLI1. The result showed that FLI1 knockdown had no effect on the accumulation of HIF‐1α protein under hypoxic conditions (Fig. 4A), indicating that FLI1 might have an effect on the transactivation of HIF‐1 target genes.

It has been demonstrated that certain ETS factor can physically interact with the alpha subunit of the HIF complex [20, 21]; we thus conducted the co‐immunoprecipitation assay to examine the protein interaction between FLI1 and HIF‐1α. As shown in Fig. 4B, anti‐FLI1 antibody (but not IgG) successfully precipitated the complex of FLI1 and HIF‐1α under hypoxia. Additionally, previous studies have indicated that the proximity (less than 60 bp) between the ETS factor binding site and the HRE may enable co‐regulation of HIF target gene transcription by ETS transcription factors and HIFs [21]; we thus analyzed the difference in base‐pair length between these two elements in the promoter region of the aforementioned HIF‐1 target genes. Interestingly, the FLI1‐dependent HIF‐1 target genes, including *BNIP3*, *PGK1*, *SLC2A1*, and *VEGFA*, have at least one putative FBS within 60 bp of a functional HRE, while the distance between the FBS and the HRE in the promoter regions of the FLI1‐independent HIF‐1 targets was as large as 100 bp (Fig. S2). To further confirm that specific co‐regulation of HIF‐1 target gene transcription by FLI1 is dependent on the proximity between the FBS and the HRE, we cloned the *BNIP3*, *VEGFA*, and *DDIT4* promoter sequences [32, 33, 34], which contain both well‐characterized HRE and putative FBSs, into individual pGL3‐Basic luciferase reporter vectors (Fig. 4C), and examined the effect of FLI1 deficiency on luciferase expression in human embryonic kidney (HEK293T) cells. Consistent with the gene expression pattern shown in Fig. 3 and S3A, FLI1 knockdown significantly reduced the hypoxia‐induced luciferase activity of *BNIP3* and *VEGFA*, but not *DDIT4*, whose FBS is far away from the core HRE (Fig. 4D).

We next performed ChIP‐qPCR assay to verify the direct interaction between FLI1 and the promoter region of FLI1‐dependent HIF‐1 target genes. A series of primer pairs covering different putative FBS in the promoter regions of *BNIP3* and *VEGFA* were deigned. As shown in Fig. 4E,F, there was a significantly enhanced interaction between FLI1 and putative FBSs in both promoter regions of *BNIP3* (FBS1: −605/−595; FBS2: −413/−404; FBS3: −350/−340) and *VEGFA* (FBS2: −917/−907) after hypoxia. Collectively, these results indicate that FLI1 may serve as a co‐regulator for HIF‐1α and selectively regulate the expression of a subset of HIF‐1 target genes by directly binding to a core promoter region containing FBS in close proximity to a functional HRE.

## Discussion

Evidence supporting an essential role for ETS transcription factors in regulating hypoxia‐inducible genes has been accumulating for the last two decades. Several ETS transcription factor family members, such as ETS1 and ELK1, have been demonstrated to specifically cooperate with HIF‐2α to activate the transcription of a number of HIF‐2 target genes under hypoxic conditions [20, 21]. In addition, previous studies have shown that other ETS family members, such as ETS2 and E74‐like ETS transcription factor 1 (ELF1), are involved in the transcription of several pro‐angiogenic genes that are preferentially regulated by HIF‐1 in endothelial cells under hypoxic conditions [37, 38], suggesting a potential link between ETS transcription factors and HIF‐1 transcriptional activity. In this study, we show that an endothelial‐specific ETS transcription factor, FLI1, is induced by hypoxia and is required for the hypoxia‐induced expression of several, but not all, HIF‐1 target genes in endothelial cells, indicating a selective regulatory role for FLI1 in activating the expression of HIF‐1 target genes under hypoxic conditions.

FLI1 is highly expressed in vascular endothelial cells and plays a critical role in early vascular development [39]. Loss of FLI1 in mice results in embryonic lethality, due in part to aberrant hemorrhaging caused by defects in blood vessel integrity during vascular development [40]. More recent studies have demonstrated that FLI1 is involved in the transcription of pro‐angiogenic genes whose expression is regulated by HIF‐1, including *ENG* and *HMOX1* [29, 30]. In the present study, we found that FLI1 is transcriptionally activated by hypoxia in endothelial cells. FLI1 knockdown significantly diminished the hypoxia‐induced expression of *VEGFA*, further confirming the essential role of FLI1 in maintaining vascular homeostasis. Furthermore, we observed that the hypoxia‐induced transcription of HIF‐1 mediated glucose metabolic genes, such as *PGK1* and *SLC2A1*, were also impaired in the absence of FLI1, indicating that FLI1 may be involved in the regulation of cellular glucose metabolism under hypoxic conditions. This was further confirmed by the reduction in glucose uptake and lactate production after FLI1 knockdown in endothelial cells under hypoxic conditions. FLI1 has been shown to regulate genes and pathways associated with cancer initiation and progression, and its abnormal expression or translocation induces various types of human cancers [28, 41]. The coregulatory role of FLI1 in promoting HIF‐1‐mediated angiogenesis and glucose utilization identified in the present study may therefore provide new mechanistic insights into tumor growth and progression to malignancy.

In addition to the aforementioned FLI1‐dependent HIF‐1 target genes, we also identified a group of HIF‐1 target genes whose transcription under hypoxic conditions was not affected by FLI1 knockdown, including *DDIT4*, *ADM*, *ANKRD37*, and *KDM3A*. This led us to investigate the molecular basis underlying selective FLI1 regulation of the expression of HIF‐1 target genes under hypoxic conditions.

Given that the distance (< 60 bp) between the ETS factor binding site and the HRE may be an important determining factor for ETS co‐regulation of HIF‐2 target genes [21], we analyzed the core promoter sequences of the eight HIF‐1 target genes mentioned above. Interestingly, there was a significant difference in the number of base pairs between the putative FBS and the functional HRE between FLI1‐dependent and FLI1‐independent HIF‐1 target genes. For example, in the promoters of the FLI1‐dependent genes *BNIP3* and *VEGFA*, there was at least one putative FBS within 60 bp of the functional HRE, while the distance between these two elements was as large as 100 bp in the FLI1‐independent HIF‐1 target gene *DDIT4*. Furthermore, a luciferase reporter assay demonstrated that FLI1 knockdown significantly impaired HIF‐1α‐induced transcription of a luciferase reporter fused to the core promoter sequence of *BNIP3* or *VEGFA*, but not *DDIT4*. Since FLI1 was shown to directly bind to the promoter regions of HIF‐1 target genes and physically interact with HIF‐1α under hypoxic conditions, it is reasonable to propose that FLI1 selectively regulates the expression of HIF‐1 target genes by interacting with HIF‐1α and enabling simultaneous binding of these two proteins to adjacent FBS and HRE elements in the promoter regions of a subset of HIF‐1 target genes.

In summary, we identified a specific ETS transcription factor, FLI1, that is involved in the transactivation of certain HIF‐1 target genes under hypoxic conditions. Together with previous reports of the essential role that other ETS factors play in HIF‐2 target gene transcription, the data from our study suggest that ETS transcription factors play a universal coregulatory role in selectively activating the expression of specific HIF‐regulated genes. Systematic dissection of the interplay between ETS transcription factors and HIFs in the future may provide more mechanistic insight into the fine‐tuning of HIF target gene expression under different hypoxic conditions or in different cell types.

## Conflict of interest

The authors declare no conflict of interest.

## Author contributions

GZ and TW conceived the study and designed the experiments. GZ, TW, and JZ contributed to the data collection. GZ and TW performed the data analysis and interpreted the results. GZ and TW wrote the manuscript. YK and LF contributed to the critical revision of the article. All authors read and approved the final manuscript.

## Supporting information

Fig. S1. Transcriptomic changes in EA.hy926 cells after exposure to hypoxic conditions.

Fig. S2. Location of the FBS and HRE elements in the promoter regions of HIF‐1 target genes.

Fig. S3. Effects of FLI1 knockdown on the expression of HIF‐1 target genes in HEK293T cells and DNA fragment detection after ultrasonic treatment for ChIP assay.

Table S1. Relevant data of RNA‐sequencing analysis. (A, B) Gene list of DEGs and upregulated HIF‐1 target genes in EA.hy926 cells under hypoxia. (C) The list of previously validated HIF‐1 target genes.

Supplementary Material

## Data Availability

The data that supports the findings of this study are presented in the main manuscript or in the supplementary material of this article. Gene list of RNA sequencing was shown in Table S1. The raw data of RNA‐seq can available from GSE176365.
