# Supplementary figures and images for: FLI1 mediates the selective expression of hypoxia‐inducible factor 1 target genes in endothelial cells under hypoxic conditions
Source: FEBS Open Bio. 2021 Jun 26;11(8):2174–85. doi: 10.1002/2211-5463.13220 (PMC8329784; doi:10.1002/2211-5463.13220)

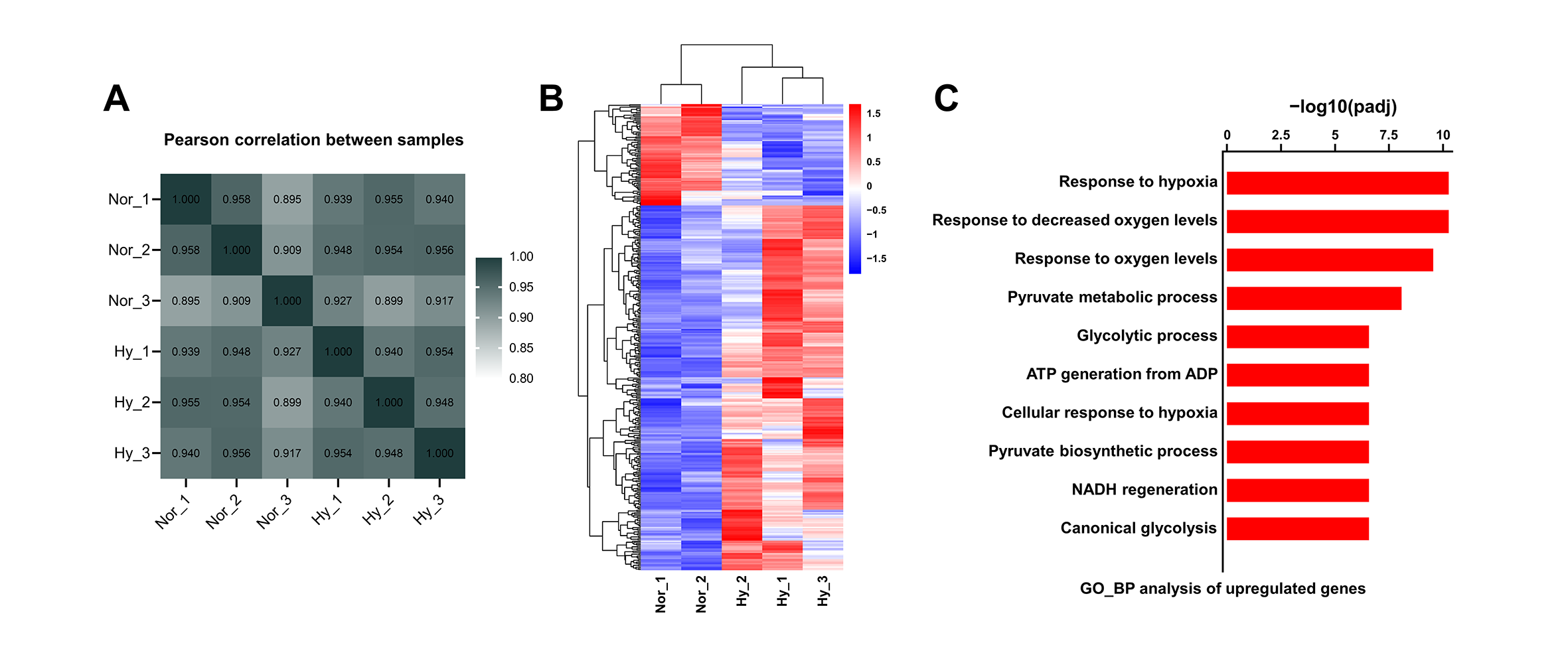

Supplement: Supplementary file 1 — Fig. S1. Transcriptomic changes in EA.hy926 cells after exposure to hypoxic conditions. [file FEB4-11-2174-s001.tif]

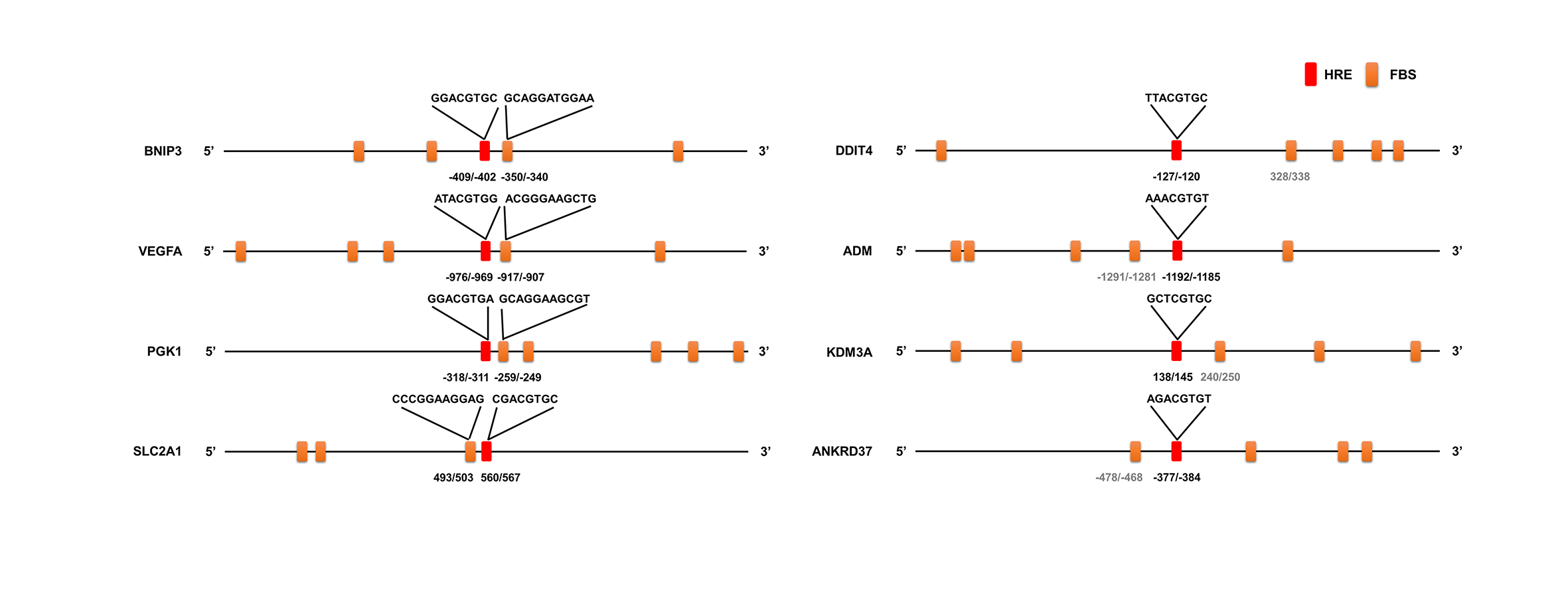

Supplement: Supplementary file 2 — Fig. S2. Location of the FBS and HRE elements in the promoter regions of HIF‐1 target genes. [file FEB4-11-2174-s002.tif]

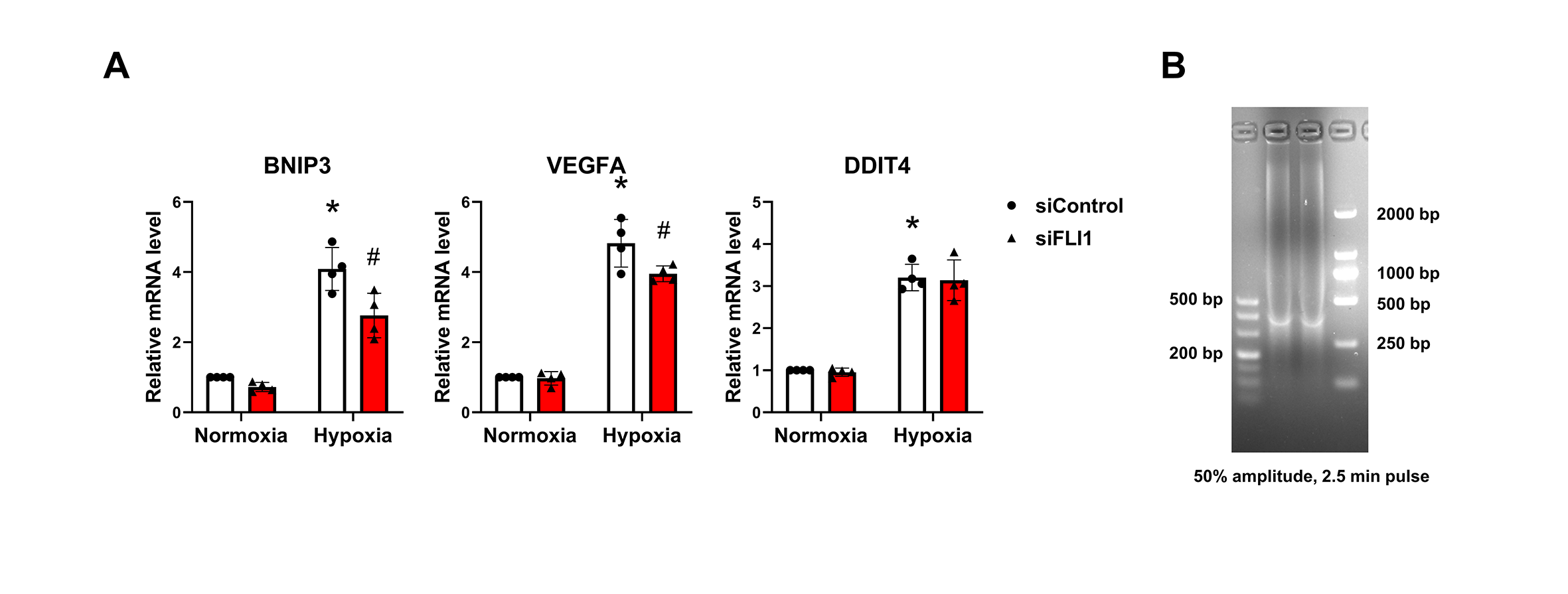

Supplement: Supplementary file 3 — Fig. S3. Effects of FLI1 knockdown on the expression of HIF‐1 target genes in HEK293T cells and DNA fragment detection after ultrasonic treatment for ChIP assay. [file FEB4-11-2174-s004.tif]
