# Supplementary material for: FLI1 mediates the selective expression of hypoxia‐inducible factor 1 target genes in endothelial cells under hypoxic conditions
Source: FEBS Open Bio. 2021 Jun 26;11(8):2174–85. doi: 10.1002/2211-5463.13220 (PMC8329784; doi:10.1002/2211-5463.13220)
Supplement: Supplementary file 5 — Supplementary Material [file FEB4-11-2174-s003.docx]

**Fig. S1.** Transcriptomic changes in EA.hy926 cells after exposure to hypoxic conditions. (A) Pearson correlation analysis of the RNA-sequencing data. (B) Heatmap of DEGs after exposure to hypoxic conditions. (C) GO_BP analysis of DEGs that were up-regulated after exposure to hypoxic conditions. Nor: normoxia group; Hy: hypoxia group.

**Fig. S2.** Location of the FBS and HRE elements in the promoter regions of HIF-1 target genes. FLI1-specific binding sites (FBSs) in the sequences flanking the functional HRE (±1000 bp) in the promoter regions of eight HIF-1 target genes were predicted using the JASPAR online database.

**Fig. S3.** Effects of FLI1 knockdown on the expression of HIF-1 target genes in HEK293T cells and DNA fragment detection after ultrasonic treatment for ChIP assay. (A) HEK293T cells were transfected with either a mismatch control siRNA (siControl) or a FLI1-specific siRNA (siFLI1) for a total of 48 h with or without the final 8 h spent under hypoxic conditions. *BNIP3*, *VEGFA,* and *DDIT4* mRNA levels were determined by RT-qPCR. (B) Analysis of interrupted DNA fragments by ultrasonic treatment of the hypoxic or normoxic cells, cultured and crosslinked by 1% formaldehyde. Data are presented as the mean ± SD, n=4. **P* < 0.05 compared with the normoxia siControl group, #*P* < 0.05 compared with the hypoxia siControl group, as determined by two-way ANOVA analysis.

**Table S1**. Relevant data of RNA-sequencing analysis. (A, B) Gene list of DEGs and upregulated HIF-1 target genes in EA.hy926 cells under hypoxia. (C) The list of previously validated HIF-1 target genes.
